# Glucomannan From *Acrocomia corumbaensis* (Bocaiuva) Attenuates Pain and Inflammation in DSS‐Induced Colitis via Mucus Barrier Protection

**DOI:** 10.1111/1440-1681.70164

**Published:** 2026-11-08

**Authors:** Marcela S. C. Brito, Anderson K. Taporosky, Katlin S. Rech, Lucas B. S. Santos, Marcelo B. Gois, Larissa F. N. de Souza, Rilton A. de Freitas, Carolina A. B. Azevedo, Carolina Mathias, Lucimara M. C. Cordeiro, Maria F. P. Werner

**Affiliations:** ^1^ Pharmacology Department Federal University of Paraná Curitiba Paraná Brazil; ^2^ Biochemistry and Molecular Biology Department Federal University of Paraná Curitiba Paraná Brazil; ^3^ Faculty of Health Sciences of the Federal University of Rondonópolis Rondonópolis Mato Grosso Brazil; ^4^ BioPol, Pharmacy Department Federal University of Paraná Curitiba Paraná Brazil; ^5^ Genetic Department Federal University of Paraná Curitiba Paraná Brazil

**Keywords:** cytokines, dextran sulphate sodium, inflammatory bowel disease, mucin, polysaccharide

## Abstract

Ulcerative colitis (UC) is a chronic inflammatory bowel disease characterized by weight loss, abdominal pain, diarrhoea and rectal bleeding. Polysaccharides have recognized protective and immunoregulatory effects in the gut. This study investigated BFS, a glucomannan extracted from bocaiuva, in dextran sulphate sodium (DSS)‐induced colitis. Male and female C57BL/6J mice received 3% DSS and BFS (1, 10, or 100 mg/kg, orally) for 7 days. Disease activity index (DAI) was monitored daily, while abdominal mechanical hypersensitivity and behavioural tests were assessed using von Frey filaments, elevated plus maze and nest‐building tests. Colons were collected for histological and biochemical analyses. BFS did not prevent DAI increase, but at 100 mg/kg reduced mechanical hypersensitivity. BFS prevented histopathological damage, inflammatory infiltration and alterations in mucosal architecture. BFS also reduced MPO, IL‐1β, TNF‐α, IFN‐γ and NFκB phosphorylation. Additionally, BFS preserved mucin content and MUC‐2 expression, with QCM‐D analysis corroborating its mucoadhesive potential. In males, BFS improved behavioural welfare parameters. Although BFS did not improve clinical signs, it preserved the mucus barrier and exerted anti‐inflammatory and analgesic effects, supporting its therapeutic potential in UC.

## 1 Introduction

Ulcerative colitis (UC) is a chronic inflammatory bowel disease clinically characterized by weight loss, abdominal pain, diarrhoea and rectal bleeding [1]. The underlying pathogenesis involves intestinal barrier disruption, marked inhibition of mucin production, epithelial damage and altered tight junction expression, which promotes microbial translocation, immune cells' activation, accelerated cytokine release, oxidative stress and chronic inflammation [2]. Despite current pharmacological, biological and surgical interventions, many patients remain refractory or experience significant adverse effects [3, 4].

Bocaiuva (*Acrocomia corumbaensis*), a native fruit of the Brazilian Cerrado, is rich in bioactive dietary fibres [5]. Its soluble fraction (BFS) is an acetylated glucomannan composed of β‐D‐1,4‐linked mannose and glucose (4.3:1 ratio; 24% acetylation; Mw = 1.82 × 10^5^ g/mol) with previously demonstrated gastroprotective effects [6]. Given the therapeutic potential of polysaccharides in UC [7], this study investigated the therapeutic potential of BFS in a murine model of DSS‐induced colitis.

## 2 Results

### 2.1 Effects of BFS on 3% DSS‐Induced Colitis

On Days 7 and 8, DSS induced weight loss of 4.5% and 13% in females, and 7% and 17% in males (Figure 1A,B), with males exhibiting a 32% greater reduction than the female DSS group. DAI scores peaked on day 8, reaching scores of 6.1 ± 0.4 and 8 ± 0.4 for females and males, respectively (Figure 1C,D). Two‐way ANOVA confirmed sex dimorphisms, with males showing a 24% higher DAI score than the female DSS group.

**FIGURE 1:**
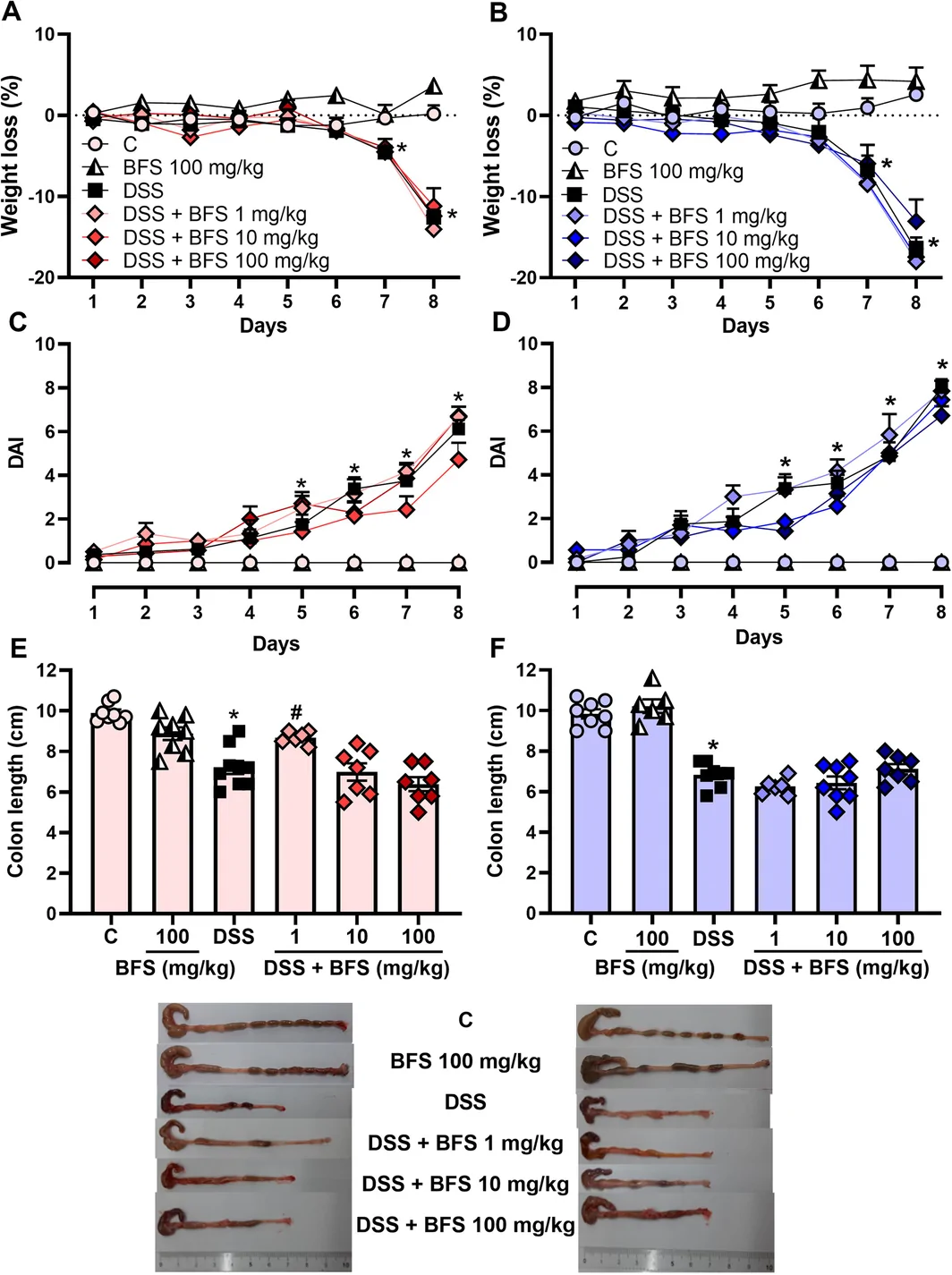
Effect of BFS on 3% DSS‐induced UC. Body weight change (A and B), DAI (disease activity index) (C and D) and colon length (E and F) on female (red) and male (blue) C57BL/6J mice. Animals were orally treated with vehicle (water, 0.1 mL/kg) or BFS (1, 10 or 100 mg/kg). Mean ± S.E.M. (*n* = 6–8). Two‐way repeated‐measures (A–D) or one‐way (E and F) ANOVA followed by Bonferroni's test. *p < 0.05 when compared to control group. # *p* < 0.05 when compared to DSS group.

DSS significantly shortened colon length in female (by 27%) and in male (by 31%) when compared to control groups (9.9 ± 0.2; 9.8 ± 0.2 cm, respectively). Particularly, BFS 1 mg/kg increased the colon length of females by 20% when compared to the DSS group (Figure 1E,F). However, BFS treatment failed to prevent these UC signals. The severity of DSS‐induced colitis was greater in males, requiring the euthanasia of two animals (one from the DSS control group and one from the BFS 1 mg/kg group) upon reaching predefined humane endpoints.

### 2.2 Effects of BFS on Abdominal Mechanical Hypersensitivity

DSS‐induced colitis increased visceral mechanical hypersensitivity, starting at day 4 and peaking at day 8. Responses were elevated in 400%, 209%, 164%, 311% and 153% in females and in 500%, 212%, 218%, 318% and 240% in males by day 8, relative to controls for the 0.008, 0.02, 0.04, 0.07 and 0.16 g filaments, respectively (12.5% ± 5.3%; 27.5% ± 6.5%; 35% ± 6.3%; 22.5% ± 8.0%; 37.5% ± 8% frequency of responses in females; 7.5% ± 3.7%; 20% ± 6.5%; 20% ± 7.6%; 27.5% ± 7.5%; 25% ± 7.3% frequency of responses in males). BFS 100 mg/kg reduced mechanical hypersensitivity to the respective filaments by 63%, 70%, 66%, 66% and 55% in females and in 62%, 22%, 48%, 51% and 36% in males compared to DSS groups (Figure 2A–J).

**FIGURE 2:**
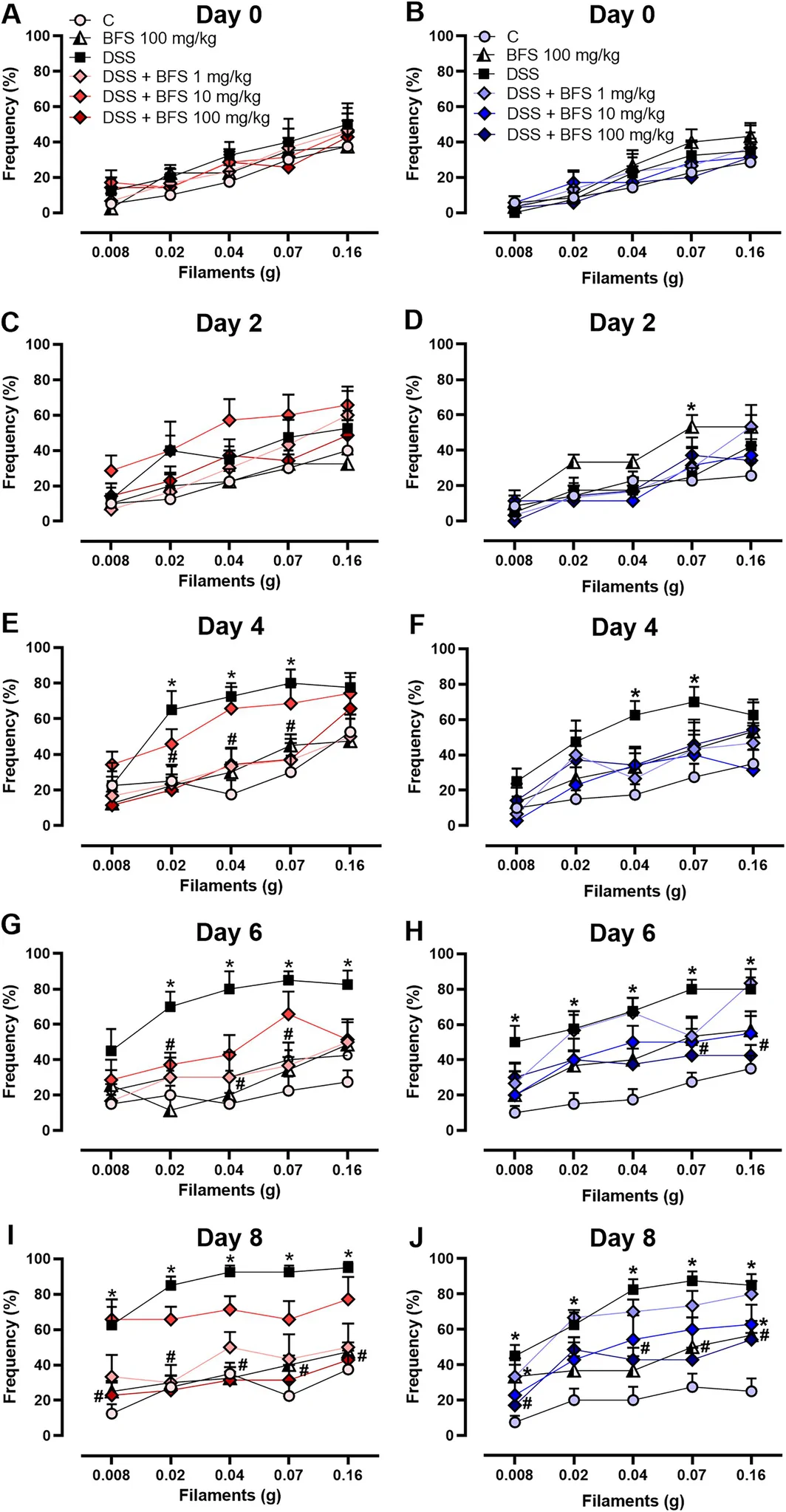
Effect of BFS on abdominal mechanical hypersensitivity (A–J) in female (red) and male (blue) mice. Animals were orally treated with vehicle (water, 0.1 mL/kg) or BFS (1, 10 or 100 mg/kg). Mean ± S.E.M. (*n* = 6–8). Two‐way ANOVA followed by Bonferroni's test. *p < 0.05 when compared to control group. #*p* < 0.05 when compared to DSS group.

### 2.3 Effects of BFS on Colon Histomorphometric Parameters

Histomorphometric analysis showed an increased submucosal thickness in DSS‐treated females and males (218% and 142%, respectively) and an increased muscular layer thickness in DSS females (68%) compared to control groups (Table 1). DSS also reduced mucosal thickness in females by 28% compared to control group. BFS (100 mg/kg) ameliorated DSS‐induced histopathological changes in females, resulting in a 35% thinner submucosa and a 39% thicker mucosa relative to DSS group.

**TABLE 1:** Histomorphometric analysis of the colon wall of female and male C57BL/6J mice receiving 3% DSS in drinking water and orally treated with vehicle (water, 0.1 mL/kg) or BFS (100 mg/kg).

Parameters (μm)^a^		Female			Male		
		C	DSS	DSS + BFS 100 mg/kg	C	DSS	DSS + BFS 100 mg/kg
Muscular	Thickness	56.6 ± 6.6	95.2 ± 7.8*	91.9 ± 15.3	109.5 ± 12.1	101.5 ± 12.1	116.5 ± 15.5
Submucosa		31.4 ± 4.1	100.0 ± 9.0*	65.0 ± 6.3**	31.5 ± 3.2	76.2 ± 6.0*	64.4 ± 7.4
Mucosa		278.1 ± 20.3	201.0 ± 11.4*	278.8 ± 7.4**	270.5 ± 12.9	252.5 ± 10.3	294.5 ± 13.8
Crypts	Depth	112.9 ± 3.1	53.4 ± 8.6*	121.9 ± 6.7**	129.5 ± 6.4	74.7 ± 6.7*	113.6 ± 6.7**
	Width	34.6 ± 1.1	19.7 ± 3.9*	31.3 ± 0.8**	32.6 ± 0.7	24.5 ± 1.6*	32.4 ± 1.4**
	Ratio	3.6 ± 0.2	1.9 ± 0.2*	4.1 ± 0.3**	4.3 ± 0.3	2.7 ± 0.2*	3.8 ± 0.3**
Enterocytes	Height	20.4 ± 0.7	16.9 ± 0.5*	19.4 ± 0.5**	21.3 ± 0.1	15.0 ± 0.3*	19.3 ± 0.8**
	Width	8.7 ± 0.5	9.1 ± 0.5	8.9 ± 0.5	8.0 ± 0.5	8.8 ± 0.4	9.1 ± 0.4
	Ratio	2.4 ± 0.1	1.9 ± 0.1*	2.3 ± 0.1**	2.8 ± 0.2	1.8 ± 0.1*	2.2 ± 0.1

^a^ Results are expressed as mean ± S.E.M (*n* = 6).

* *p* < 0.05 when compared to control group.

** *p* < 0.05 when compared to DSS group.

DSS also reduced intestinal crypt depth and width by 53% and 43% in females, and by 42% and 25% in males, as well as the depth‐to‐width ratio (by 48% and 38%, respectively), compared to control groups. BFS 100 mg/kg prevented these alterations, increasing crypt depth, width and depth‐to‐width ratio by 118%, 58% and 120% in females, and by 52%, 32% and 42% in males, respectively, compared to DSS groups (Table 1, Figure 3A).

**FIGURE 3:**
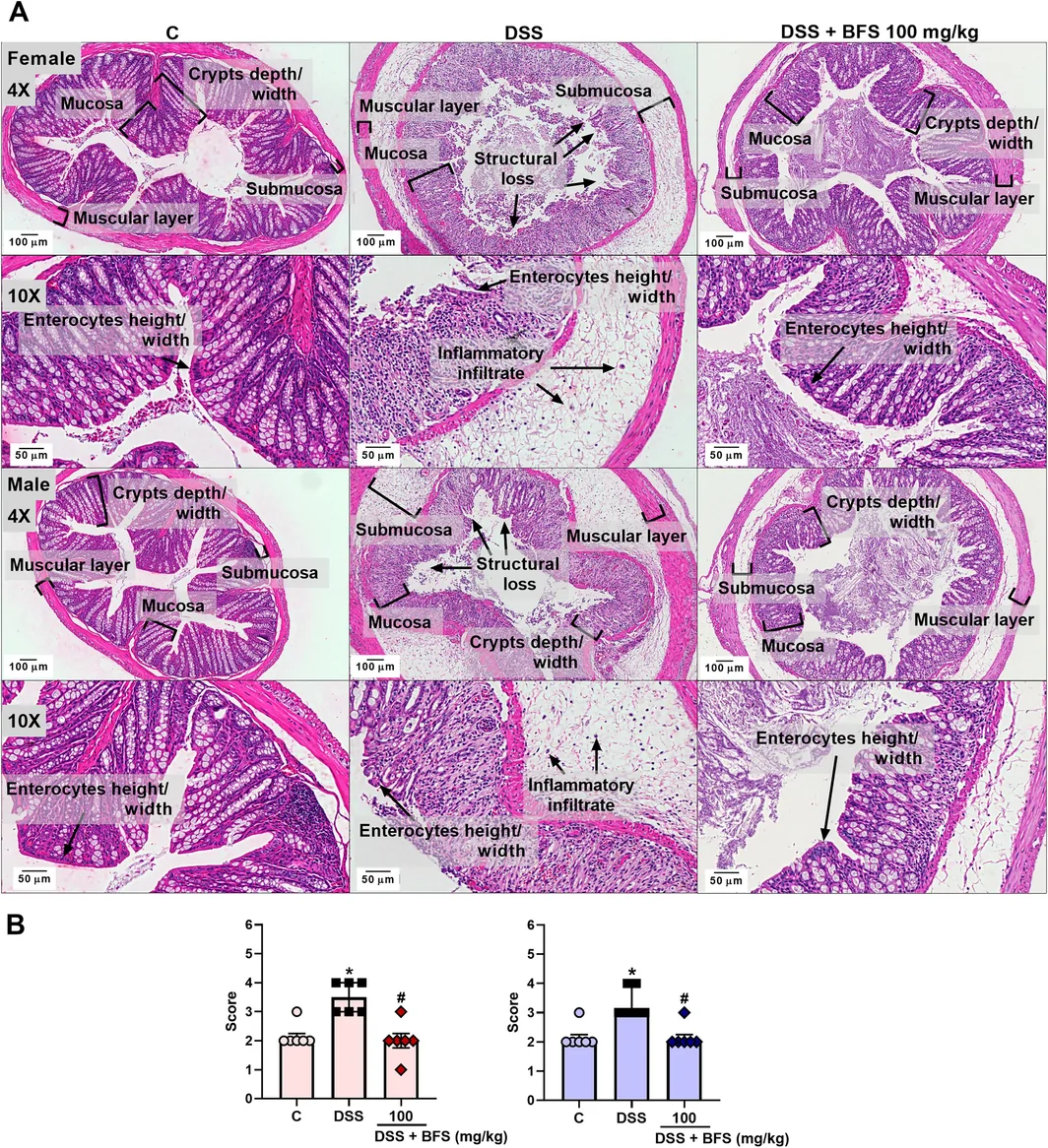
Effect of BFS on colon histology in female (red) and male (blue) mice, (×4, bars = 100 μm and ×10, bars = 50 μm (A)). Animals were orally treated with vehicle (water, 0.1 mL/kg) or BFS (100 mg/kg). Median ± interquartile range (*n* = 6). Kruskal‐Wallis followed by Dunn's test. *p < 0.05 when compared to control group. #*p* < 0.05 when compared to DSS group (B).

In addition, DSS groups showed a decrease in enterocytes height and height‐to‐width ratio in 17% and 20% in females and in 29% and 36% in males when compared to control groups. BFS 100 mg/kg prevented this decrease, displaying 15% and 21% higher enterocytes and ratio in females and 28% higher enterocytes in males compared to DSS groups (Table 1, Figure 3A).

### 2.4 Effects of BFS on Colon Histopathology

Colons of mice exposed to DSS exhibited severe damage compared with control groups. Histopathological scores increased by 75% in females and 50% in males compared to control groups (both 2.0 scores). BFS 100 mg/kg attenuated inflammatory cells infiltration and preserved histoarchitecture, reducing damage scores by 42% in females and 33% in males, respectively, when compared to DSS groups (Figure 3A,B).

### 2.5 Effects of BFS on Colonic Inflammation

DSS increased colonic MPO levels by 218% in females and 143% in males when compared to control groups (9.2 ± 1.2; 10.6 ± 1.6 O.D./mg of protein). BFS 100 mg/kg reduced MPO levels in 63% in females and 73% in males (Figure 4A,B).

**FIGURE 4:**
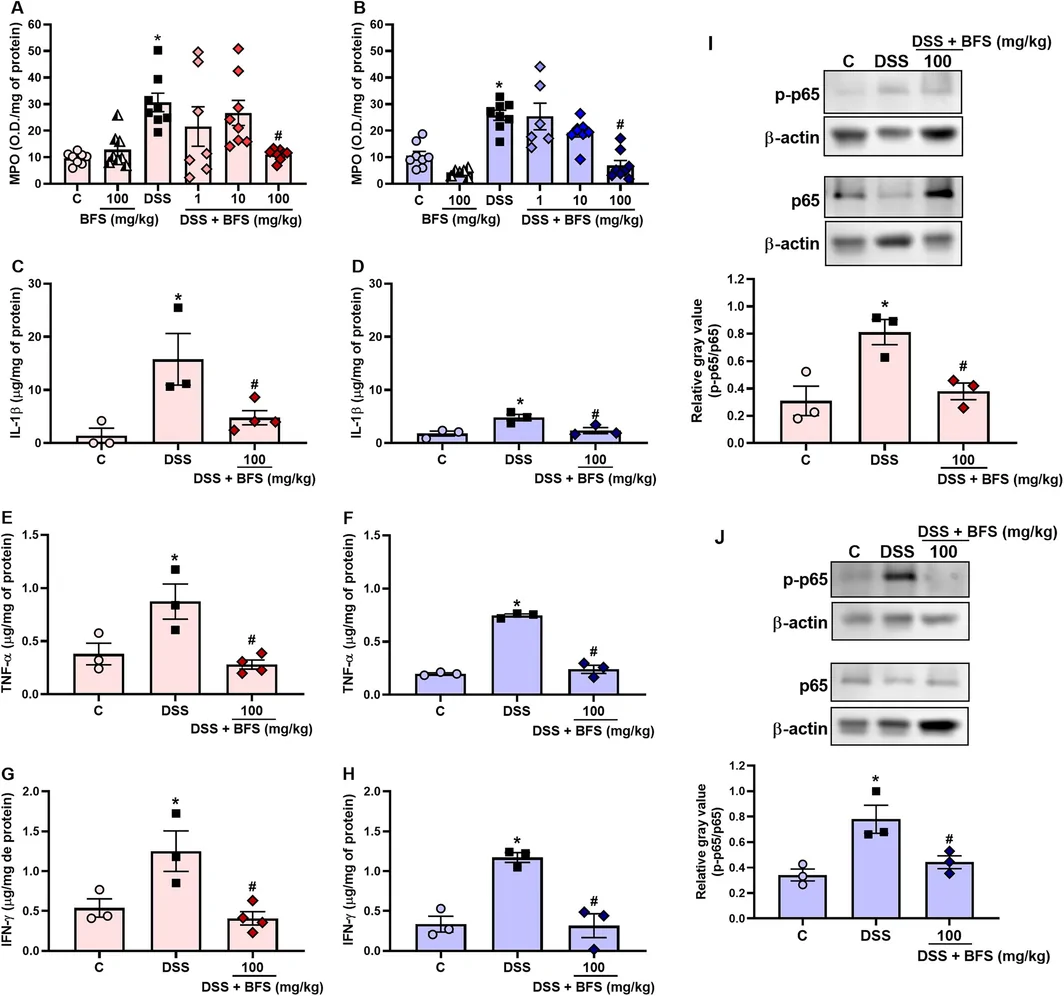
Effects of BFS on MPO (A and B), IL‐1β (C and D), TNF‐α (E and F), IFN‐γ (G and H) and p‐p65/p65 (I and J) levels in the colon of female (red) and male (blue) mice. The uncropped and full‐length blots are presented in Figure S2. Animals were orally treated with vehicle (water, 0.1 mL/kg) or BFS (1, 10 or 100 mg/kg). Mean ± S.E.M. (A and B: *n* = 6–8; C–J: *n* = 3–4). One‐way ANOVA followed by Bonferroni's test. *p < 0.05 when compared to control group. #*p* < 0.05 when compared to DSS group.

The proinflammatory cytokines IL‐1β, TNF‐α and IFN‐γ were increased by DSS in 1022%, 130% and 132% in females and by 170%, 278% and 249% in males, in comparison to control groups (1.4 ± 1.4; 0.4 ± 0.1; 0.5 ± 0.1; 1.8 ± 0.5; 0.2 ± 0.01 and 0.3 ± 0.1 μg/mg of protein) (Figure 4C–H). BFS 100 mg/kg reduced the levels of IL‐1β, TNF‐α and IFN‐γ by 70%, 68% and 67% in females and in 51%, 68% and 73% in males, compared to DSS groups. IL‐10 levels showed no significant differences among treatment groups for either sex (Figure S1A,B).

DSS increased the levels of phospho‐NFkB p65/NFkB p65 ratio by 162% in females and 128% in males, relative to control groups (0.3 ± 0.1; 0.3 ± 0.05, respectively) (Figures 4I,J and S2). BFS 100 mg/kg decreased these ratios by 53% in females and 43% in males.

### 2.6 Effects of BFS on Colonic Mucus

DSS treatment significantly decreased acid mucin levels (by 37% in females and 59% in males) and neutral mucin levels (by 34% in females and 63% in males) compared to their respective control groups (172 743 ± 8756; 153 027 ± 13 946; 186 923 ± 20 224 and 135 245 ± 17 754 pixels/field, respectively). BFS prevented the DSS‐induced mucin loss, and at 100 mg/kg, it restored acid and neutral mucin levels to 62% and 84% in females, and 115% and 195% in males, compared to DSS groups (Figure 5A,B). DSS significantly reduced MUC‐2 expression by 89% in females and 53% in males, compared to control groups (101 578; 58 493 pixels/field, respectively). In contrast, BFS 100 mg/kg increased MUC‐2 immunoexpression by 415% in females and 113% in males, compared to the DSS groups (Figure 6A).

**FIGURE 5:**
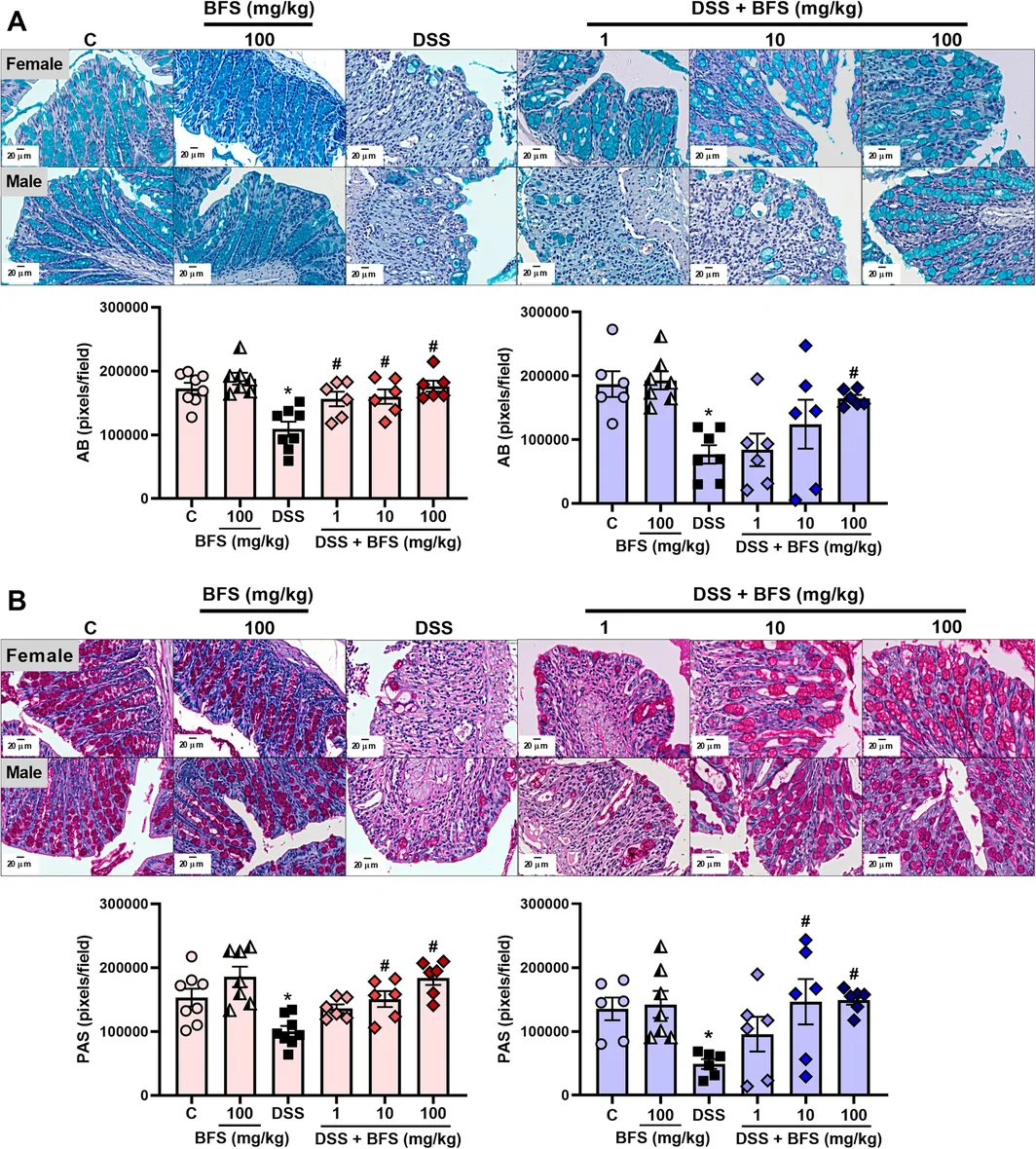
Effects of BFS on acid (A) and neutral (B) mucin levels in the colon of female (red) and male (blue) mice, (×20, bars = 20 μm). Animals were orally treated with vehicle (water, 0.1 mL/kg) or BFS (1, 10 or 100 mg/kg). Mean ± S.E.M. (*n* = 6–8). One‐way ANOVA followed by Bonferroni's test. *p < 0.05 when compared to control group. #*p* < 0.05 when compared to DSS group.

**FIGURE 6:**
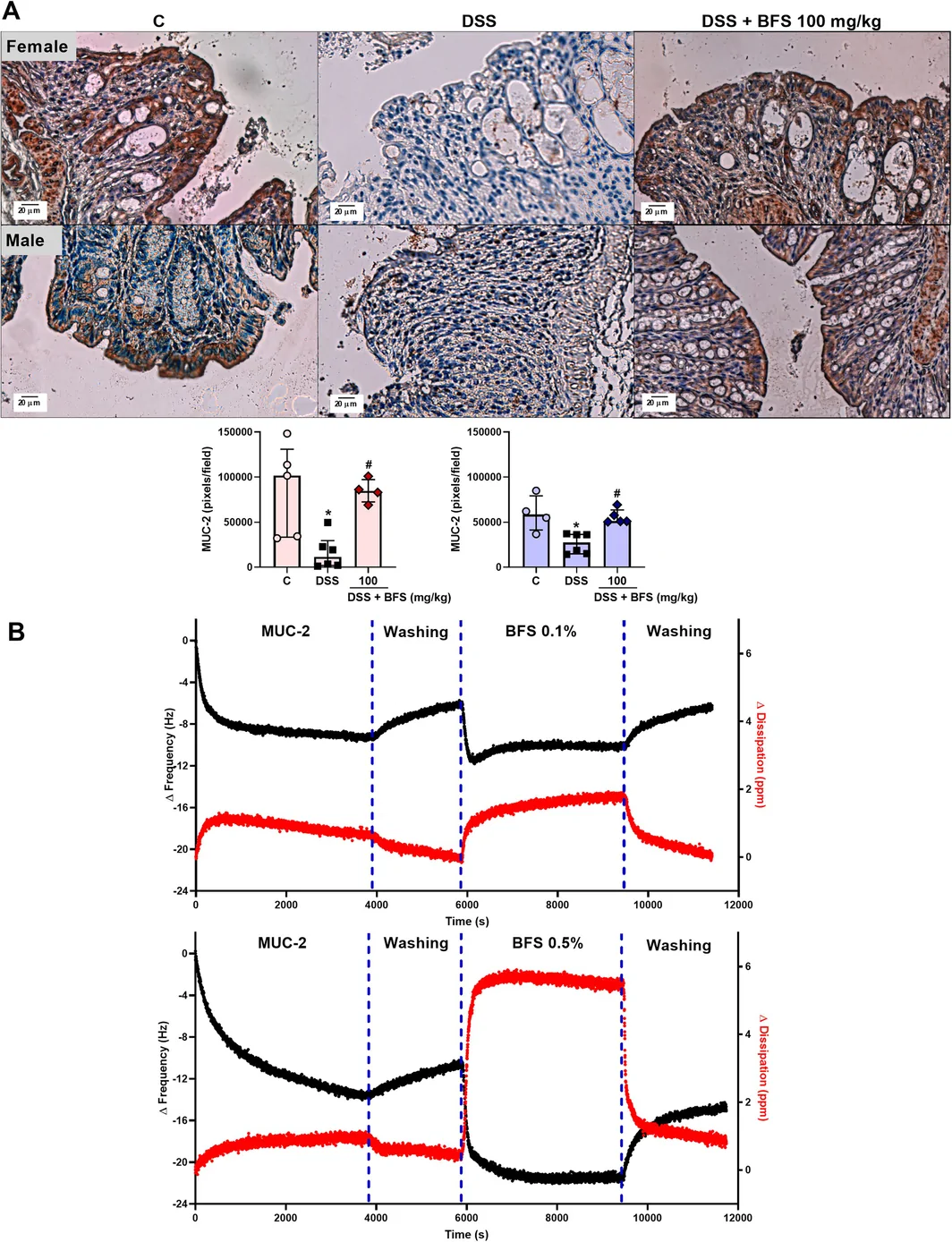
Effect of BFS on MUC‐2 levels (A) in the colon of female (red) and male (blue) mice, (×20, bars = 20 μm). Animals were orally treated with vehicle (water, 0.1 mL/kg) or BFS (100 mg/kg). Median ± interquartile range (*n* = 4–6). Kruskal‐Wallis followed by Dunn's test. *p < 0.05 when compared to control group. #*p* < 0.05 when compared to DSS group. BFS interaction with MUC‐2 on QCM‐D (B). Mean of 3rd, 5th, 7th, 9th and 11th harmonics.

### 2.7 BFS Adhesion on Mucin Surface

In QCM‐D BFS 0.1% analysis, the introduction of MUC‐2 led to a decrease in frequency to −9.2 Hz, accompanied by an increase in dissipation to around 0.6 ppm at 3915 s (Figure 6B). After washing, frequency increased to −6.4 Hz and dissipation decreased to approximately 0 at 5870 s, indicating the formation of a viscoelastic layer. Following the addition of 0.1% BFS, a drop in frequency was observed, reaching −9.9 Hz, and dissipation increased to 1.7 ppm at 9485 s, indicating polymer adsorption and the formation of a thick and hydrated layer. After the rinsing, frequency increased to −6.6 Hz and dissipation decreased to 0.1 ppm at 11410 s, indicating a reversible interaction between BFS 0.1% and MUC‐2.

In BFS 0.5% analysis, MUC‐2 addition caused a frequency reduction to −13.4 Hz and an increase in dissipation to 0.9 ppm at 3865 s. After washing, frequency increased to −10.9 Hz and dissipation decreased to approximately 0.6 ppm at 5895 s, corroborating the formation of a viscoelastic layer. Subsequently, BFS 0.5% addition further intensified the frequency decrease to around −21.1 Hz, while dissipation increased to 5.5 ppm at 9379 s, indicating the formation of a more compact and rigid adsorbed film. Remarkably, after rinsing, frequency increased to −15.1 and dissipation decreased to 0.8 ppm at 11739 s, indicating that part of BFS 0.5% remained adhered to MUC‐2.

### 2.8 Effects of BFS on PCNA and HSP70 Expression

DSS treatment reduced PCNA colonic levels by 64% in females and 68% in males relative to control groups (134 958 ± 5799 and 132 362 ± 3619 pixels/field, respectively), an effect unaffected by BFS (Figure S3A). DSS treatment significantly upregulated colonic HSP70 expression, increasing levels by 156% in females and 168% in males, compared to control groups (40 470 ± 1853 and 37 188 ± 2270 pixels/field). BFS 100 mg/kg attenuated this effect in females, reducing HSP70 levels by 41% compared to the DSS group (Figure S3B).

### 2.9 Effects of BFS on Animal Welfare

EPM evaluation demonstrated a significant effect of treatment on the time spent in the open arms across both sexes [*F*(4, 32) = 3.168; *p* < 0.05 females; *F*(4, 31) = 8.545; *p* < 0.05 males], signalling an anxiety‐like phenotype and reduced welfare. Female DSS‐treated mice spent 50% less time in the open arms compared to control groups (6.9 ± 0.8 s). Males exhibited reductions in time spent in open arms (by 84%), open arm entries (by 74%) [*F*(4, 31) = 7.161; *p* < 0.05], closed arm entries (by 29%) [*H*(4, 31) = 9.530; *p* < 0.05] and head dips (by 67%) [*H*(4, 31) = 9.193; *p* < 0.05] relative to controls (8.4 ± 1.1 s; 4.4 ± 0.6 entries; 17 entries; 3 dips, respectively). Male mice treated with BFS 100 mg/kg displayed a 307% increase in time spent in open arms and a 294% increase in open arm entries (Figure 7A–H).

**FIGURE 7:**
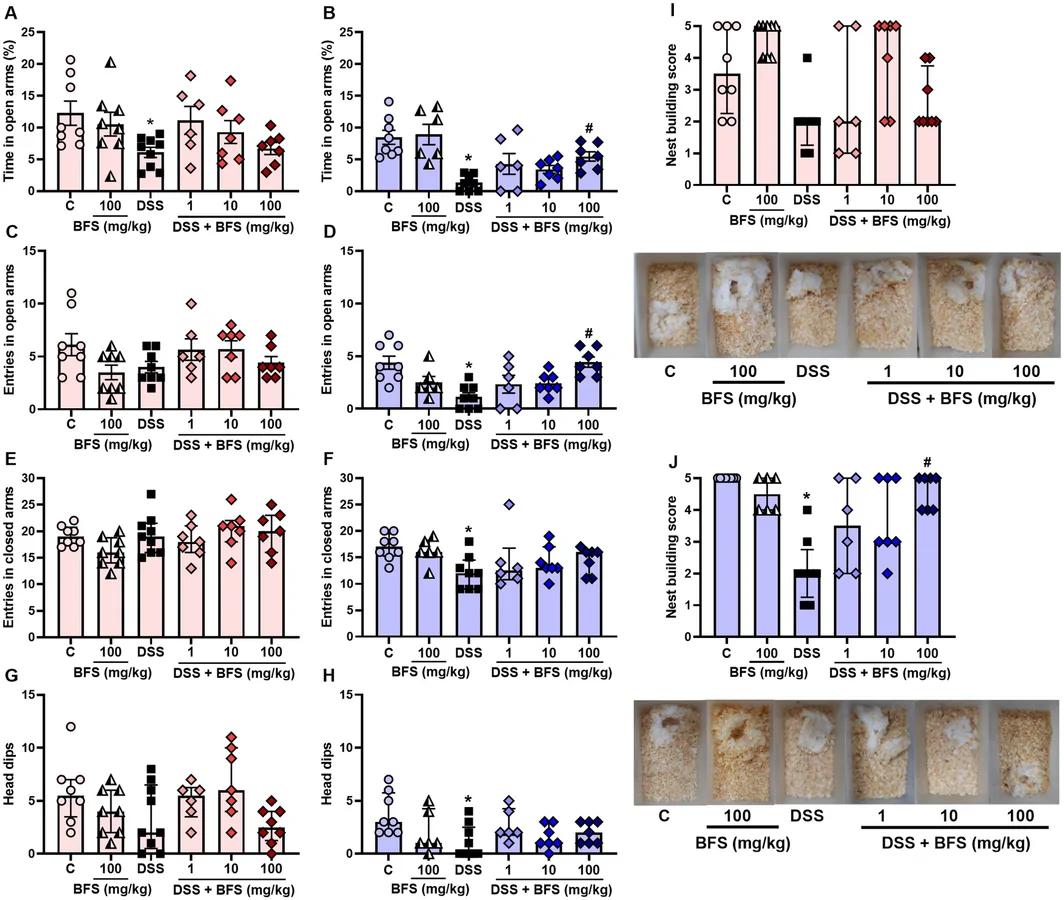
Effects of BFS on EPM (A–H) and nest building (I and J) parameters in female (red) and male (blue) mice. Animals were orally treated with vehicle (water, 0.1 mL/kg) or BFS (1, 10 or 100 mg/kg). Mean ± S.E.M. (A–D) or median ± interquartile range (E–J) (*n* = 6–8). One‐way ANOVA followed by Bonferroni's test (A–D) or Kruskal‐Wallis followed by Dunn's test (E–J).*p < 0.05 when compared to control group. #*p* < 0.05 when compared to DSS group.

Regarding nesting activity, no significant differences were observed between female groups. DSS treatment reduced nesting scores in males by 57% [*H*(4, 31) = 20.16; *p* < 0.05] compared to controls (5.0 score), whereas BFS 100 mg/kg restored scores by 115% compared to the DSS group (Figure 7I,J).

### 2.10 Estrous Cycle Influence

No estrous cycle phase‐dependent variations were detected in female DAI, nest building, or abdominal mechanical hypersensitivity measured at Day 8 (Figsure S4A,B,D). Statistical differences were only found on MPO levels, where BFS 1 mg/kg proestrus females had levels 272% higher than estrous and 761% higher than metestrus females (12.8 ± 7.4; 5.5) (Figure S4C).

## 3 Discussion

Colitis was effectively induced in both sexes, as indicated by increased DAI from Day 5 and consistent clinical signs. BFS did not improve DAI at any dose. DSS‐induced colon shortening was partially prevented only by BFS 1 mg/kg in females, suggesting an isolated effect. Abdominal pain was the only clinical sign prevented by BFS. In sharp contrast, BFS attenuated histological damage, neutrophil infiltration, cytokines' increase and NFκB phosphorylation and depletion of acidic and neutral mucins, while preserving MUC‐2 expression. These findings indicate anti‐inflammatory, analgesic and mucosal barrier‐protective effects.

The dissociation between clinical symptoms and tissue protection may reflect the specificity of BFS action. Clinical parameters such as weight loss and diarrhoea involve systemic factors, including fluid balance and metabolism, whereas BFS appears to act primarily at the mucosal level, preserving epithelial integrity and mucus barrier function [8, 9, 10, 11]. QCM‐D analysis demonstrated that BFS interacts with MUC‐2, confirming its mucoadhesive potential. At 0.5%, BFS promoted a marked decrease in frequency and increase in dissipation, with partial retention after rinsing, consistent with the formation of a hydrated viscoelastic layer on the mucin surface. This interaction may underlie the preservation of mucin content and MUC‐2 expression observed in vivo, thereby mitigating inflammation without immediately resolving acute clinical symptoms. These findings suggest that mucoadhesion is necessary but not sufficient to achieve full clinical efficacy, which probably also depends on residence time under physiological conditions, polymer diffusion through the mucus layer, dosage, treatment duration and modulation of additional inflammatory pathways [12, 13, 14, 15, 16].

Besides mucoadhesion, polysaccharide can modulate immune responses through toll‐like receptors (TLRs) interactions [17, 18, 19], a mechanism dependent on structural characteristics such as mannose:glucose (Man:Glc) ratio, molecular weight and degree of acetylation [20, 21, 22]. Glucomannans with lower Man:Glc ratios and higher molecular weights exhibit pronounced anti‐inflammatory effects [23], which accounts for the intermediate efficacy observed for BFS (4.3:1; 182 kDa). Moreover, acetylation further modulates function, while higher acetylation may enhance immune recognition and solubility [20, 21, 22], deacetylation improves gel formation and mucosal adhesion, favouring barrier protection [24]. Thus, BFS effects likely result from a balance between immunological and physicochemical properties.

BFS reduced IL‐1β, TNF‐α, IFN‐γ, MPO and HSP70 contents, and reduced NFκB phosphorylation, consistent with its anti‐inflammatory properties [2], however, it failed to reduce HSP70 in males. These differences may reflect sex‐specific hormonal and microbial influences, as well as greater disease severity limiting responsiveness [25]. Notably, no differences were observed in IL‐10 levels, despite its established role in immune regulation [26, 27].

Behavioural assays showed that DSS impaired well‐being, reflecting inflammation‐induced sickness behaviour [28]. Although not specific to intestinal inflammation, elevated plus maze and nest‐building tests provide complementary information on functional impairment [28, 29, 30, 31]. Attenuation of behavioural deficits by BFS suggests additional benefits related to pain modulation and well‐being, supporting gut–brain axis involvement.

Behavioural changes were predominant in males, indicating sex‐dependent responses to visceral pain. Similarly, reduced exploratory behaviour was observed exclusively in DSS‐treated male mice [23]. Conversely, some studies failed to detect nesting alterations [24], suggesting limited sensitivity in the C57BL/6J strain. Alternatively, digging behaviour offers greater sensitivity and directly correlates with histopathology [32].

In this study, male mice exhibited greater UC severity than females across weight loss, DAI and welfare indicators. Although direct comparisons remain limited, previous studies have also reported increased susceptibility in male C57BL/6J mice [32]. These dimorphisms are likely multifactorial, driven by sex hormones that modulate both immune responses and intestinal homeostasis [33], with oestrogens enhancing epithelial barrier integrity and limiting pro‐inflammatory signalling, whereas androgens exert less protective, context‐dependent effects [34, 35]. Additionally, sex‐dependent microbiota variations influence UC progression by affecting mucosal immunity, epithelial function and metabolite production [25, 36]. Together, these factors likely underlie the increased male susceptibility, underscoring sex as a critical biological variable.

In summary, BFS failed to attenuate clinical severity but preserved mucin content, prevented histopathological damage, reduced inflammatory mediators and partially improved welfare. These findings suggest that BFS acts primarily as a barrier‐protective and anti‐inflammatory agent rather than a broad modulator of clinical symptoms, supporting its potential as a complementary therapy targeting mucosal integrity.

## 4 Methods

### 4.1 Polysaccharide Isolation and Characterization

Glucomannan from bocaiuva (*Acrocomia corumbaensis*) was isolated and characterized at the Carbohydrate Chemistry Laboratory, Federal University of Paraná. Fruits were harvested ripe in Corumbá, MS, Brazil. Bocaiuva flour (BF) was obtained by pulp dehydration (40°C, 24 h), and sieving (20 mesh). BF was defatted (methanol: chloroform 1:1, Soxhlet) and air‐dried.

Dietary fibres were extracted by the enzymatic–gravimetric method (AOAC 991.43). After centrifugation (3500 rpm, 25 min, 4°C), the supernatant (soluble fibres) was concentrated, precipitated with ethanol (3:1), centrifuged, dialyzed (12–14 kDa, 48 h) and lyophilized to obtain BFS [6]. Structural characterization was performed by NMR (^1^H, ^13^C, HSQC; Bruker 400 and 600 MHz). Acetylation was determined by the Hestrin method [6].

### 4.2 Animals

Male and female C57BL/6J mice (6–9 weeks, 17–27 g) were obtained from the Federal University of Paraná vivarium. Animals were housed (≤ 10/cage) in polypropylene boxes (39 × 34 × 16 cm), with a galvanized grid under controlled conditions (22°C ± 2°C, 12 h light/dark cycle) with wood shaving bedding, food (Nuvi‐Lab CR‐1, Quimtia S/A, Brazil) and filtered water *ad libitum*. Environmental enrichment included cardboard paper rolls, disposable mask hammocks and polysulfone flats (Alesco).

Procedures were approved by the Ethics Committee on the Use of Animals of the Biological Sciences Sector of the Federal University of Paraná (CEUA‐BIO‐UFPR, approval number 1628) and followed Brazilian Guidelines for the Care and Use of Animals for Scientific and Educational Purposes (DBCA) and ARRIVE.

### 4.3 Experimental Design

Animals were allocated according to baseline abdominal mechanical sensitivity. The testing apparatus consisted of individual acrylic boxes divided into compartments, on an elevated apparatus with a metal mesh floor. Basal visceral hypersensitivity was assessed by applying von Frey filaments (0.008, 0.02, 0.04, 0.07 and 0.16 g; Aesthesio, Bioseb) to the abdominal region. Each filament was applied five times per animal. Hypersensitivity responses were defined as rapid abdominal withdrawal, licking or jumping, and these behaviours were quantified as response frequency. Male and female mice were then separately assigned (*n* = 6–8/group) to: Control (C), BFS (100 mg/kg), DSS or DSS + BFS (1, 10 or 100 mg/kg) groups.

### 4.4 Colitis Induction and Preventive Treatment

3% DSS (Dextran Products, Scarborough, Ontario, Canada, Mw = 40 kDa) was added in drinking water for 7 days [37, 38, 39, 40]. During this period, mice received vehicle (water) or BFS (1, 10 or 100 mg/kg) once a day, through oral gavage. The experimental endpoint was defined as a 15 point score on the CEUA‐BIO‐UFPR animal well‐being scale (Table S1). Animals reaching this endpoint were euthanized by inhalation of isoflurane followed by cervical dislocation.

### 4.5 Disease Activity Index (DAI)

DAI was calculated daily as the sum of body weight loss (0: < 1%, 1: 1%–5%, 2: 6%–10%, 3: 11%–15%, 4: > 15%), stool consistency (0: solid, 2: loose, 4: liquid) and blood in the stool (0: negative, 2: positive, determined by the Meyer‐Johannessen reaction) [17, 18].

### 4.6 Colon Length

On Day 8, mice were euthanized as previously described. Excised colons were measured for length using a ruler and subsequently sectioned for further analysis.

### 4.7 Abdominal Mechanical Hypersensitivity

Abdominal hypersensitivity was measured on days 0, 2, 4, 6 and 8, as described above.

### 4.8 Histomorphometric Analysis

Distal colon sections were fixed in 4% formalin for 24 h, dehydrated with ethanol and xylol, embedded in paraffin and cut in 5 μm sections with a microtome. Tissue sections were deparaffinized and subjected to haematoxylin and eosin (H&E) staining. Histomorphometric analyses were performed using Image‐Pro Plus software (Media Cybernetics, USA) on images captured with a digital camera (Nikon DS‐Fi3, 5.9 MP) attached to an optical microscope (Nikon Eclipse TS2R). The thickness of the colonic layers and the dimensions of the crypts and enterocytes were assessed, and the crypt depth‐to‐width and enterocyte height‐to‐width ratios were calculated, with values approaching 1 indicating a loss of normal colonic histoarchitecture [41].

### 4.9 Histopathological Score

The same H&E‐stained sections were examined blindly under a light microscope (Nikon Eclipse TS2R) by a specialist to assess inflammatory cell infiltration and loss of intestinal histoarchitecture to previously established criteria (Table 2) [41, 42, 43].

### 4.10 Myeloperoxidase (MPO) Assay

Median colon samples were stored at 4°C and homogenized in 80 mM sodium phosphate buffer containing 0.5% hexadecyltrimethylammonium bromide (HTAB). Following centrifugation (11 000 rpm, 20 min, 4°C), supernatants were analysed for MPO levels using the method of Bradley et al. modified by De Young et al. [44, 45]. Absorbance was read at 620 nm on a Synergy HT microplate reader (Biotek, USA). Results were normalized to total protein, measured by the Bradford assay (Bio‐Rad, Hercules, CA, USA), using a bovine serum albumin standard curve (62.5–1000 g/mL) [17, 18].

### 4.11 Quantification of Colonic Cytokines

Median‐distal colon sections were kept in RIPA buffer (1 M Tris–HCl pH 7.5, 5 M NaCl, 0.5 M EDTA and 1% Triton‐X, 4°C) supplemented with 1% protease and phosphatase inhibitor cocktails (Sigma Chemicals, MO, USA), homogenized and centrifuged at 9000 rpm for 20 min. Supernatants were used to quantify IL‐1β, TNF‐α, IFN‐γ and IL‐10 levels using ELISA kits (Peprotech EC Ltd., London UK) according to the manufacturer's recommendations. Total protein content of the samples was determined as described in MPO assay.

### 4.12 NFκB p65 Western Blot Analysis

Equal amounts of protein (75 μg), from the cytokine supernatants were separated using 12% SDS‐PAGE and transferred onto nitrocellulose membranes. Membranes were blocked for 1 h in Tris‐buffered saline containing 5% BSA and 0.3% Tween 20. After washing with TBS‐Tween 20 (0.05%), membranes were incubated overnight with the primary antibodies anti‐β‐actin or anti‐NFkB p65 (both rabbit polyclonal IgG) or anti‐Phospho‐NFkB p65 (Ser536) (rabbit monoclonal IgG), all 1:1000 Invitrogen, Thermo Fisher Scientific at 4°C. Primary antibodies were detected using a peroxidase conjugated secondary antibody (goat anti‐rabbit IgG, 1:10000, Invitrogen, Thermo Fisher Scientific) and visualized by SuperSignal West Pico PLUS (ThermoFisher Scientific). Band intensities were quantified densitometrically using Image Studio Lite 5.0 (Lincoln, NE, USA), normalized to β‐actin and expressed as the relative p‐p65/p65 ratio.

### 4.13 Mucins Quantification

Colon sections were stained with Alcian blue (AB, pH 2.5) or Periodic acid–Schiff (PAS), respectively [17]. Slides were photographed with a microscope (Axio Imager Z2, Carl Zeiss) using a 20× objective linked to a MetaViewer scanner (version 2.0.10, MetaSystems, Germany). Colour intensity was quantified with Image J 1.30v software (National Institute of Health, USA).

### 4.14 Immunohistochemical Analysis

Additional colon sections were subjected to immunohistochemistry for mucin 2 (MUC‐2), proliferating cell nuclear antigen (PCNA) and heat shock protein 70 (HSP70). The slides were processed using primary antibodies anti‐MUC‐2 (rabbit polyclonal IgG, 1:100, Invitrogen, Thermo Fisher Scientific), anti‐PCNA or anti‐HSP70 (goat polyclonal IgG, 1:100, Santa Cruz Biotechnology), followed by peroxidase‐conjugated secondary antibody (goat anti‐rabbit, Invitrogen, Thermo Fisher Scientific or rabbit anti‐goat Santa Cruz Biotechnology, both 1:100). Peroxidase activity was detected by chromogen diaminobenzidine (DAB Substrate Kit, BD Pharmingen) staining and counterstaining with Mayer's haematoxylin [17]. Slides were photographed and colour intensity was quantified as described before.

### 4.15 BFS Adhesion to Isolated Mucin via Quartz Crystal Microbalance With Dissipation Monitoring (QCM‐D)

To evaluate BFS adhesion to isolated MUC‐2, QCM‐D was performed using a QCM‐D E4 (Biolin Scientific AB, Gothenburg, Sweden), containing a quartz piezoelectric sensor. This sensor recorded changes in frequency and dissipation at the fundamental frequency of 4.95 MHz. BFS solutions were prepared at concentrations of 0.1% and 0.5% (w/v). All measurements were conducted in continuous buffer flow with a flow rate of 0.1 mL/min at 37°C ± 0.1°C using a peristaltic pump (ISM9335C, Ismatec, Zurich, Switzerland). The results were recorded at the crystal frequencies [16].

### 4.16 Elevated Plus Maze (EPM)

On Day 7, 1 h after oral treatment, mice were placed individually in the centre of an EPM apparatus (30 cm × 5 cm arms, connected in the middle by a 5 × 5 cm open centre, with closed arm walls with 15 cm height), and recorded for 5 min. The apparatus was cleaned with a 10% ethanol solution between animals to eliminate olfactory cues [46].

### 4.17 Nest Building Behaviour

On Day 7, 1 h before the dark cycle, animals were individually placed in polypropylene boxes (30 × 19 × 12 cm) with galvanized grid and wood shaving bedding. Animals were kept overnight with food and water *ad libitum* and a piece of cotton (~95 g). At the onset of the light cycle, nests were photographed and scored by a blinded evaluator. Nesting behaviour was assessed as follows: 1—untouched cotton; 2—partially shredded, 50% or more remained intact; 3—well‐shredded but dispersed, without a delimited nest; 4—well‐shredded with a delimited flat nest; 5—well‐shredded with a delimited crater‐shaped nest [47].

### 4.18 Vaginal Cytology

On Day 8, estrous cycle phases were evaluated to assess potential hormonal influences on experimental outcomes (nest‐building, DAI, abdominal mechanical hypersensitivity, and MPO). Vaginal smears were collected, analysed and categorized in proestrus, estrus, metestrus or diestrus according to Vilela et al. [48].

### 4.19 Statistical Analysis

All data was assessed for normality using the Shapiro–Wilk test. Parametric data was analysed by one‐ or two‐way ANOVA, followed by Bonferroni's post hoc test. Nonparametric data was analysed using the Kruskal‐Wallis test, followed by Dunn's post hoc test. All analyses were performed in GraphPad Prism software (version 8.0.1, GraphPad Software Inc.), and statistical significance was defined as *p* < 0.05.

## Author Contributions

M.S.C.B. and M.F.P.W.: writing – original draft, review and editing, methodology, investigation, formal analysis, data curation, conceptualization. A.K.T., K.S.R., L.B.S.S., M.B.G., L.F.N.d.S., R.A.d.F., C.A.B.A., C.M. and L.M.C.C.: methodology, investigation.

## Funding

This work was supported by Conselho Nacional de Desenvolvimento Científico e Tecnológico (CNPq 308816/2023‐4, 313982/2020‐1), Coordenação de Aperfeiçoamento de Pessoal de Nível Superior (CAPES, Finance Code 001).

## Conflicts of Interest

The authors declare no conflicts of interest.

## Supporting information


**Figure S1:** Effect of BFS on IL‐10 levels (A‐B) in the colon of female (red) and male (blue) mice. Animals were orally treated with vehicle (water, 0.1 mL/kg) or BFS (100 mg/kg). Mean ± S.E.M. (*n* = 3–4). One‐way ANOVA followed by Bonferroni's test.
**Figure S2:** Representative full‐length Western blot membranes showing p‐p65, p65 and β‐actin in female and male mice. The EA.hy926 cell lysate was included as a positive control for protein detection. Left panels show membranes imaged under visible light to visualize the non‐chemiluminescent molecular weight marker (blue), whereas the corresponding right panels show the chemiluminescence images used for protein detection.
**Figure S3:** Effect of BFS on PCNA (A) and HSP70 (B) levels in the colon of female (red) and male (blue) mice, (x 20, bars = 20 μm). Animals were orally treated with vehicle (water, 0.1 mL/kg) or BFS (100 mg/kg). Median ± interquartile range (A) or mean ± S.E.M. (B) (*n* = 4–6). Kruskal‐Wallis followed by Dunn's test (A) or one‐way ANOVA followed by Bonferroni's test (B). * *p* < 0.05 when compared to control group. # *p* < 0.05 when compared to DSS group.
**Figure S4:** Effects of estrous cycle on day 8 DAI (A), nest building (B) and MPO levels (C) and day 8 abdominal mechanical hypersensitivity (D). Mice were orally treated with vehicle (water, 0.1 mL/kg) or BFS (1, 10 or 100 mg/kg). Mean ± S.E.M. (*n* = 6–8). Two‐way ANOVA followed by Bonferroni's test. * *p* < 0.05 when compared to proestrus subgroup.
**Table S1:** Parameters and scores for animal well‐being evaluation.
**Table S2:** Histopathological score.

## Data Availability

Data described in the manuscript, code book and analytic code will be made available upon request pending application and approval.
