# Supplementary material for: Glucomannan From Acrocomia corumbaensis (Bocaiuva) Attenuates Pain and Inflammation in DSS‐Induced Colitis via Mucus Barrier Protection
Source: Clin Exp Pharmacol Physiol. 2026 Nov 8;53(11):e70164. doi: 10.1111/1440-1681.70164 (PMC13745779; doi:10.1111/1440-1681.70164)
Supplement: Supplementary file 1 [file CEP-53-0-s001.docx]

**Supplementary Figure 1**

**Supplementary Figure 1.** Effect of BFS on IL-10 levels (A-B) in the colon of female (red) and male (blue) mice. Animals were orally treated with vehicle (water, 0.1 mL/kg) or BFS (100 mg/kg). Mean ± S.E.M. (n=3-4). One-way ANOVA followed by Bonferroni’s test.

**Supplementary Figure 2**

**Supplementary Figure 2.** Representative full-length Western blot membranes showing p-p65, p65 and β-actin in female and male mice. The EA.hy926 cell lysate was included as a positive control for protein detection. Left panels show membranes imaged under visible light to visualize the non-chemiluminescent molecular weight marker (blue), whereas the corresponding right panels show the chemiluminescence images used for protein detection.

**Supplementary Figure 3**

**Supplementary Figure 3.** Effect of BFS on PCNA (A) and HSP70 (B) levels in the colon of female (red) and male (blue) mice, (x 20, bars = 20 µm). Animals were orally treated with vehicle (water, 0.1 mL/kg) or BFS (100 mg/kg). Median ± interquartile range (A) or mean ± S.E.M. (B) (n=4-6). Kruskal-Wallis followed by Dunn’s test (A) or one-way ANOVA followed by Bonferroni’s test (B). * p < 0.05 when compared to control group. # p < 0.05 when compared to DSS group.

**Supplementary Figure 4**

**Supplementary Figure 4.** Effects of estrous cycle on day 8 DAI (A), nest building (B) and MPO levels (C) and day 8 abdominal mechanical hypersensitivity (D). Mice were orally treated with vehicle (water, 0.1 mL/kg) or BFS (1, 10 or 100 mg/kg). Mean ± S.E.M. (n=6-8). Two-way ANOVA followed by Bonferroni’s test. * p < 0.05 when compared to proestrus subgroup.

**Supplementary Table 1.** Parameters and scores for animal well-being evaluation.

| **Score^1^** | | | | |
| --- | --- | --- | --- | --- |
| **Parameter^1^** | **1** | **2** | **3** | **4** |
| Spontaneous behavior | Walking, running, eating, drinking | Intermediate degree | Intermediate degree | Imobile, inactive, prostrated |
| Posture | Normal posture while standing or moving | Intermediate degree | Intermediate degree | Bent over, crouched, lying down |
| Breathing | Regular and comfortable movements | Intermediate degree | Intermediate degree | Irregular movements, forced breathing |
| Fur condition | Clean and bright | Intermediate degree | Intermediate degree | Dirty, disheveled, piloerection |
| Eyes | Clean, bright, open | Intermediate degree | Intermediate degree | Closed, presence of secretion, opaque |
| Body condition | Good muscular coverage | Intermediate degree | Intermediate degree | Emaciated, withered flanks, distended abdomen |

^1^CEUA/BIO UFPR

**Supplementary Table 2.** Histopathological score.

| **Category** | **Inflammatory cell infiltrate** | | |  | **Intestinal histoarchitecture** | | |
| --- | --- | --- | --- | --- | --- | --- | --- |
| **Criterion** | **Severity** | **Extent** | **Score 1** |  | **Epithelial changes** | **Mucosal histoarchitecture** | **Score 2** |
| **Definition** | mild | mucosa | 1 |  | focal erosions | - | 1 |
|  | moderate | mucosa and submucosa | 2 |  | erosions | ± focal ulcerations | 2 |
|  | marked | transmural | 3 |  | marked erosions | extended ulcerations ± granulation tissue ± pseudopolyps | 3 |
| **Sum of scores 1 and 2** | | | | | | | 0-6 |

^1^Score 0: normal
